# Combined, Sequential Intravenous and Intra-Arterial Chemotherapy (Bridge Chemotherapy) for Young Infants with Retinoblastoma

**DOI:** 10.1371/journal.pone.0044322

**Published:** 2012-09-18

**Authors:** Y. Pierre Gobin, Ira J. Dunkel, Brian P. Marr, Jasmine H. Francis, Scott E. Brodie, David H. Abramson

**Affiliations:** 1 Service of Interventional Neuroradiology, Departments of Neurosurgery Neurology and Radiology, Weill Cornell Medical College of New York Presbyterian Hospital, New York, New York, United States of America; 2 Departments of Pediatrics, Memorial Sloan Kettering Cancer Center and Weill Cornell Medical College, New York, New York, United States of America; 3 Ophthalmic Oncology Service, Memorial Sloan Kettering Cancer Center, and Department of Ophthalmology, Weill Cornell Medical College, New York, New York, United States of America; 4 Department of Ophthalmology, Mount Sinai School of Medicine, New York, New York, United States of America; Massachusetts Eye & Ear Infirmary, Harvard Medical School, United States of America

## Abstract

**Background:**

Intra-arterial (IA) chemotherapy has more risks of procedural complications in neonates and young infants. For these reasons, we have developed a strategy of bridge intravenous single agent chemotherapy to postpone IA chemotherapy in these children

**Procedure:**

Neonates and young infants with retinoblastoma who required chemotherapy were treated with systemic carboplatin chemotherapy (18.7 mg/kg IV every 3–4 weeks) until they reached the age of 3 months and a weight of 6 Kg. If necessary, IA chemotherapy was subsequently performed at 4 weeks intervals. Efficacy was judged by tumor regression on ophthalmological examination. Retinal toxicity was judged by electroretinography.

**Results:**

Eleven children (19 eyes) were treated. All patients are alive and no patient has developed metastatic disease or second malignancies (mean follow-up 27 months, range 9–46 months). Intravenous carboplatin (median 2 cycles, range 1–5) combined with cryotherapy and laser was given to all children. This was effective for five eyes, which did not require IA chemotherapy. IA chemotherapy was administered to 14 eyes (median 3.5 cycles per eye, range 1 to 6). No radiation therapy was required. The Kaplan Meier estimate of ocular radiation-free survival was 94.7% at one year (95% confidence interval 68.1–99.2%). One eye was enucleated due to tumor progression. ERG showed no deterioration of retinal function.

**Conclusion:**

Bridge IV-IA chemotherapy was feasible and safe, and is a promising strategy to treat retinoblastoma in neonates and young infants.

## Introduction

In neonates and young infants, retinoblastomas are often bilateral, multiple and occur most often in the posterior pole (in or near the macula), where preserving central vision is especially challenging [1], [2]. Treatment of young infants (in the first three months of life) has included a mix of laser and or cryotherapy if the tumors are small, and occasionally plaque brachytherapy, but in the past larger tumors often required external beam irradiation. Though patient and ocular salvage were good [3], short and long term complications from radiating these very young children were worrisome. In addition to the development of permanent facial abnormalities, second, non-ocular cancers developed and they occurred at the highest rate in children radiated in the first year of life [4], [5]. As a result, clinicians abandoned radiation as primary management and switched to initial management with systemic chemotherapy for such children treated in the first year of life. While this approach has been replicated with success worldwide, insufficient efficacy and concerns about short and long-term toxicity of intravenous chemotherapy have led our group to intra-arterial chemotherapy by selective infusion through the ophthalmic artery [6]. At our center, we now routinely use intra-arterial (IA) chemotherapy for the treatment of advanced intra-ocular retinoblastoma that cannot be cured by thermo-ablation or cryotherapy alone [7]–[12]. However, we hesitate to use IA chemotherapy in young infants (less than 3 months of age) because of the potential risk of complications from catheterizing small arteries. Although Yamane reported performing IA chemotherapy in a patient as young as one month and 5 days in his series of 187 patients [13], no other center has treated children this young: the youngest patient in the series of 38 patients reported by Venturi et al. was a 7-month old [14], an 11-month old in the 14 patients reported by Muen et al. [15], a 4-month old in the 17 patients reported by Shields et al. [16], a 6-month old in the 15 patients reported by Peterson et al. [17], and a 10-month old in the 13 patients reported by Munier et al. [18]. None of these authors reported on the management of retinoblastoma in neonates and young infants, which is why we think it useful to report our experience: we used intravenous chemotherapy with one or several cycles of single agent carboplatin to postpone IA chemotherapy until children reach the age of 3 months and a weight of 6 Kg, a technique that we call bridge IV-IA chemotherapy.

## Methods

The institutional review board of Weill Cornell Medical College has approved this study. Written informed consent was obtained from the parents, carers or guardians on the behalf of all the children involved in this study. Following a (previously reported) case of failed catheterization of the ophthalmic artery in a one month-old infant [11], we decided in July 2008 to postpone IA chemotherapy in neonates and young infants. Neonates and young infants with large intra-ocular retinoblastoma were treated with intravenous chemotherapy using carboplatin at a dose of 18.7 mg/kg over one hour every 3–4 weeks depending on adequate blood count recovery and ophthalmic examinations.

Each ophthalmic examination was performed (every 3–4 weeks) under general anesthesia and included indirect ophthalmoscopy, fundus photography, ophthalmic ultrasonography, and electroretinography as a surrogate for measurement of visual acuity in these very young children [7]. For the electroretinography, the amplitude of the response to the 30-Hz flicker was taken as the primary measure of retinal function. Electroretinography measures were classified according to the following scale: 0: flat; 0.1 µV–25 µV: poor; 25.1 µV–50 µV: fair; 50.1 µV–75 µV: good; 75.1 µV–100 µV: very good; >100 µV: excellent. During the examinations, laser ablation and cryotherapy were performed as needed: as a curative treatment for small tumors and applied to large tumors with the goal of increasing the permeability of the blood-retina barrier prior to IA chemotherapy, if the retina was not detached.

Once the child had reached 3 months of age and a weight of 6 kg, IA chemotherapy was considered and was performed if tumor regression was insufficient and if we judged that continuing intravenous chemotherapy with local treatments would not be curative. In such cases, IA chemotherapy was performed on the affected eye(s) every 4 weeks, usually the day following ophthalmic examination under anesthesia.

We have previously described the technique and results of IA chemotherapy in detail [6]–[11]. Briefly, IA chemotherapy was performed as an outpatient procedure under general anesthesia with endotracheal intubation. One femoral artery (alternatively right and left) was punctured and a 4F (1.3 mm outer diameter) arterial sheath was placed into the femoral artery. Anticoagulation was obtained with intravenous injection of 70 IU/Kg of heparin. Using X-ray guidance, a microcatheter (1.5F or 0.5 mm distal diameter) was advanced into the internal carotid artery and placed at the ostium of the ophthalmic artery, where the chemotherapy drug(s) was injected over 20–30 minutes. If the ophthalmic artery was difficult to catheterize, selective injection was performed using two alternate techniques: the first technique was by catheterization of the orbital branch of the middle meningeal artery, the second technique was by using a balloon to temporarily occlude flow into the distal carotid artery while injecting the drug below the balloon to direct blood flow into the ophthalmic artery [11], [13]. After the procedure, the catheter and sheath were removed and hemostasis of the femoral artery was obtained with manual compression. The child was awakened and observed for 5 hours before discharge. A complete blood count was performed about 9 days later.

Three chemotherapy drugs were used: melphalan, topotecan, and carboplatin. Melphalan was the drug of first choice, at an ocular dose of 2.5 mg in 6–7 Kg and 3 mg in >7 Kg infants. Melphalan may induce neutropenia when given at a dose exceeding 0.4 mg/kg of body weight, which is why a dose of 2.5 to 3 mg per eye cannot not be given during the same procedure to both eyes in infants (the total dose of melphalan would be 5–6 mg and for a 6–7 Kg infant highly toxic). Carboplatin, ocular dose 25–30 mg, was given as an alternative to melphalan when both eyes needed IA chemotherapy at the same time, or was added to melphalan in case of insufficient tumor regression in unilateral treatments. Topotecan, ocular dose ranging from 0.3 to 0.5 mg, was not used alone but given in addition to melphalan and/or carboplatin.

From a prospective database of all retinoblastoma patients seen in our center, we selected the cases referred to us in the first 3 months of life and in whom we decided to use bridge IV-IA chemotherapy. We included one foreign patient who had intravenous chemotherapy performed according to our bridge protocol close to home. We did not include patients referred to us after having failed therapies elsewhere.

## Results

From August 2008 till October 2011 we treated 11 children (19 eyes) with the bridge IV-IA technique. Table 1 summarizes their demographics, treatment, and results.

**Table 1:** Results of bridge IV-IA chemotherapy in retinoblastoma.

N	Sex	Uni/Bi	Age at Starting IVC	No IVC cycles	Age & weight at First IAC	Side	Classification: RE group/ICRB	No IAC cycles	Other Treatment	First-Last ERG	Response: Tumor regression - seeds regression	Follow-up (months)
1	F	U	2 months	4	5 months & 7 Kg	OD	Vb/D	4	enucleation	good-good	regression type 1 - recurrence of vitreous seeds	8
2	F	U	4 months	1	5 months & 7 Kg	OD	IIb/B	2	none	excellent- excellent	regression type 4 - no seeds	42
3	M	B	3 months	2	5 months & 9 Kg	OD	IIIa/B	0	cryo	excellent-excellent	regression type 4 - no seeds	44
						OS	IIa/B	3+1 *	laser	excellent-excellent	regressions type 3 & 4 - no seeds	44
4	M	B	1 month	5	6 months & 9 Kg	OD	Ib/B	2	laser	excellent-very good	regression type 4 - no seeds	46
						OS	Ib/A	0	laser	excellent-excellent	regression type 1 - no seeds	46
5	M	B	1 month	2	3 months & 7.5 Kg	OD	Vb/D	5	cryo+laser	poor-fair	regression type 3 – disap-pearance of vitreous seeds	31
						OS	IIIa/A	0	laser	good-excellent	regression type 4 - no seeds	31
6	M	B	1 month	2	3.5 months & 7 Kg	OD	IIIa/C	6	laser	excellent-very good	regression type 2 - no seeds	20
						OS	Vb/D	3	laser	very good-very good	regression type 1 - calcified seeds	20
7	M	B	1 month	2	3 months & 6 Kg	OD	Ib/B	2	laser	fair-very good	regression type 4 - no seeds	19
						OS	IIb/C	3	laser	fair-good	regressions type 3 & 4 - no seeds	19
8	F	B	0.5 month	4	3 months & 5 Kg	OD	Va/D	1	laser	flat-fair	regression type 1 – calcified subretinal seeds	17
						OS	Ia/B	0	laser	poor-good	regression type 4 - no seeds	17
9	F	B	1 month	3	4 months & 6 Kg	OD	IVa/B	0	laser	fair-fair	regression type 4 - no seeds	16
						OS	Vb/D	5	cryo+laser	poor-poor	regressions type 1 & 4 – calcified vitreous seeds	16
10	F	B	2 months	1	3 months & 7 Kg	OD	Vb/E	0	enucleation at diagnosis	n/a	n/a	n/a
						OS	Va/E	4	laser	good-good	regression type 3 – no seeds	10
11	M	B	2 months	2	4 months & 7 Kg	OD	IVa/C	5	laser	good-excellent	regressions type 1,3,4 – no seeds	9
						OS	Va/E	5	laser	poor-fair	regression type 1 - no seeds	9

* One additional intra-arterial chemotherapy procedure was performed for a recurrence after 3 years. ERG: Electroretinography. ICRB: International Classification of Retinoblastoma. IVC: Intravenous chemotherapy. IAC: Intra-arterial chemotherapy. RE: Reese-Ellsworth classification of retinoblastoma.

Tumor regression pattern: Type 0: disappearance; Type 1: highly calcified; Type 2: fish flesh (no vascularity and no growth); Type 3: mixed types 1 and 2; Type 4: white scar.

### Demographics

There were 5 females and 6 males. Retinoblastoma was diagnosed before 3 months in ten patients and at 4 months in one; this last child was treated with the bridge technique because her weight was less than 6 kg. Retinoblastoma was bilateral in nine patients and unilateral in two. Four of the 11 children had a positive family history including one of the unilateral cases. The 19 eyes were classified in the Reese-Ellsworth classification Group I-III in 10, Group IV in 2 and Group V in 7; in the International Classification of Retinoblastoma they were classified Group A in 2, B in 7, C in 3, D in 5, and E in 2. Each patient had at least one Group B or worse eye necessitating chemotherapy.

### Treatment

One eye (Patient #11) was enucleated at diagnosis (prior to chemotherapy) in a child with bilateral retinoblastoma for chemosis and proptosis, and is not included in the analysis. One eye (Patient #1) was enucleated due to failed catheterization and progression of disease.

Intravenous chemotherapy: the 11 patients (19 eyes) received a median of 2 cycles of carboplatin (range 1 to 5 cycles) at a dose of 18.7 mg/kg delivered every 3–4 weeks.

IA chemotherapy was performed in 14 eyes of 11 patients (bilateral in 3 cases), median 3.5 cycles per eye (range 1 to 6), and 4 cycles per patient (range 1 to 6). There were 51 IA attempts at chemotherapy infusions, 50 (98%) were successful: 38 were performed using the ophthalmic artery (OA), 6 the middle meningeal artery (MMA), and 6 using temporary balloon occlusion of the internal carotid artery (balloon). The only catheterization failure was the fourth procedure in patient #1. During the first three procedures, the ophthalmic artery was large because of an anatomical variant (a large middle meningeal artery coming off the ophthalmic artery). This eye had multiple vitreous seeds that were not responding well to IA chemotherapy, and we attributed the lack of response to the diversion of the chemotherapy drug in the middle meningeal artery. To compensate for this, during the 3^rd^ IA chemotherapy procedure we blocked permanently the middle meningeal artery, so that all drug injected into the ophthalmic artery would stay intra-ocular; unfortunately, after embolization the ophthalmic artery shrunk so much to adapt to the newly reduced flow conditions that we were unable to catheterize the reduced ophthalmic artery during the fourth procedure; the eye was enucleated.

The 50 successful IA infusions were performed with melphalan only in 24 cases, carboplatin only in 5, melphalan plus topotecan in 10, carboplatin plus topotecan in 6, melphalan plus carboplatin plus topotecan in 5.

### Complications

Complications of intravenous carboplatin: Of the 28 cycles, there were 12 episodes of Grade 3 and 4 episodes of Grade 4 toxicity. One patient required an injection of granulocyte colony-stimulating factor, no patient required a blood product transfusion.

Complications of IA chemotherapy: Procedural complications included one allergic reaction to iodine contrast that was prevented during subsequent procedures with standard premedication; one mild bronchospasm during the catheterization of the internal carotid artery; one transient pulse loss on the side of the femoral puncture that was treated with further heparinization until the pulses re-appeared after 3 hours (this child was admitted overnight). Hematologic toxicity consisted of four episodes of Grade 4 neutropenia (one patient received granulocyte colony-stimulating factor, another was admitted for rehydration and antibiotics), four episodes of Grade 3 neutropenia, one Grade 3 anemia (one patient received a red blood cell transfusion) and one Grade 3 thrombocytopenia. Ocular complications were all transient and included transient loss of the medial eyelashes in one case, ptosis in one, and frontal erythema in the cutaneous distribution of the ophthalmic artery in three (all associated with injection of melphalan).

### Clinical results

**Figure 1:**
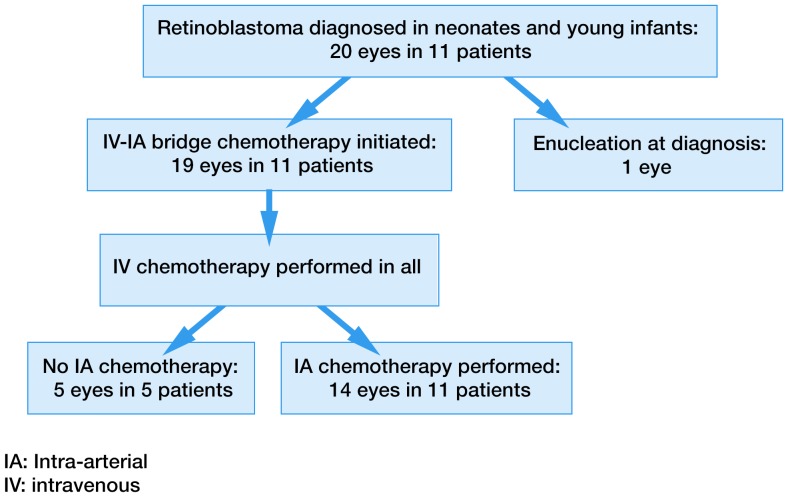
Treatment flow of Bridge IV-IA chemotherapy.

**Figure 2:**
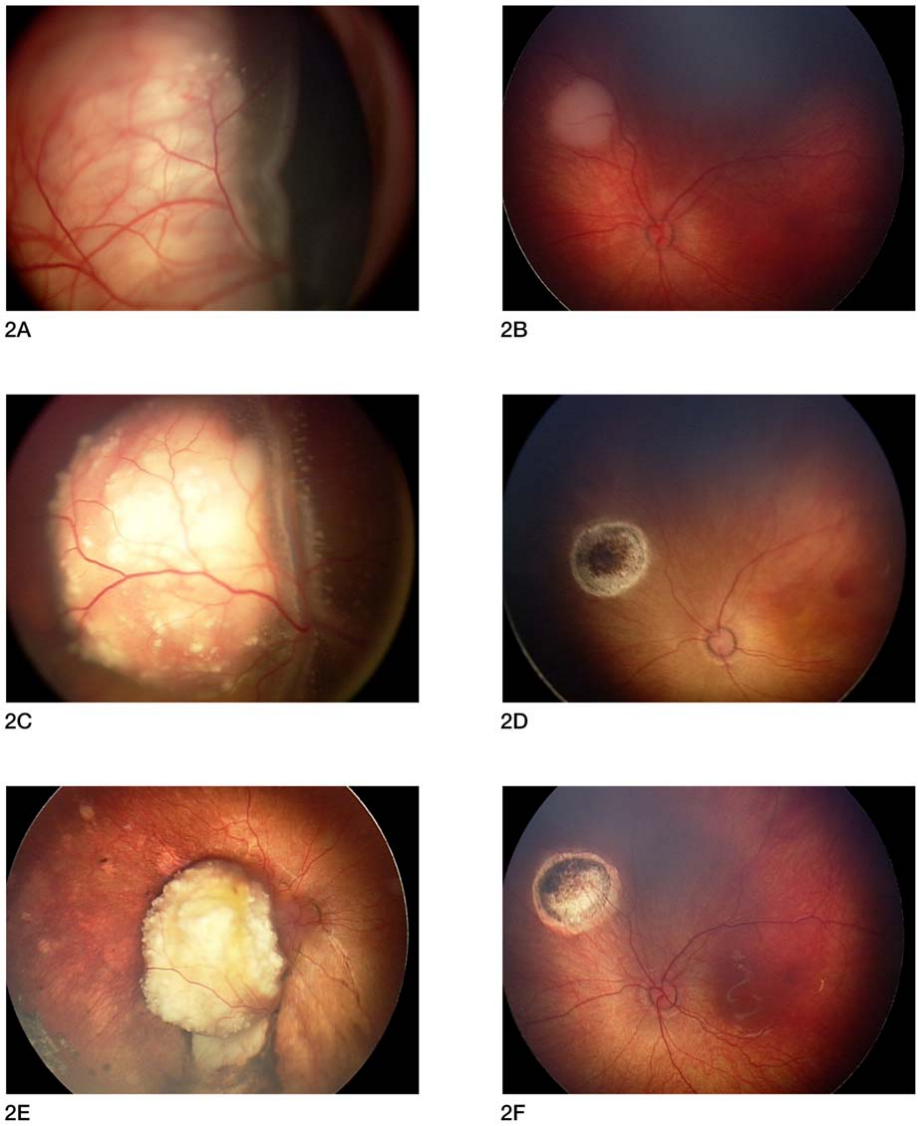
Two-week old female with bilateral retinoblastoma treated with bridge IV-IA chemotherapy. Figure 2A & 2B: Fundus photographs at initial examination (2 weeks of age). 2A: right eye: large tumor with total retinal detachment. The eye was classified Reese-Ellsworth group 5A and International classification of Retinoblastoma Group D. 2B: left eye: small tumor. The eye was classified Reese-Ellsworth group 1A and International classification of Retinoblastoma Group B. Figure 2C & 2D: Fundus photographs after 4 cycles of intravenous (IV) carboplatin chemotherapy (4 months of age). 2C: right eye: the tumor has partially regressed, the retina is still totally detached and subretinal seeds are visible. 2D: left eye: complete tumor regression with laser treatment. Figure 2E & 2F: Fundus photographs at 9 months of age, after one right ophthalmic artery IA (intra-arterial) chemotherapy performed at 4 months of age. 2E: right eye: the tumor has shrunk and calcified (Type 1 regression), with disappearance of the subretinal seeds. The retina has reattached. Final electroretinogram (ERG) was fair. 2F: left eye: white scar (Type 4 regression). Final ERG was good.

All patients are alive and none have developed extra-ocular disease or a second cancer at a mean follow-up of 27 months from diagnosis (range 9–46 months). All 11 patients (19 eyes) have completed the bridge IV-IA chemotherapy protocol. Results are detailed in Table 1 and patient flow is explained in Figure 1. Intravenous carboplatin with local treatment (cryotherapy and laser) controlled tumor growth in all 19 eyes; this treatment was sufficient for complete tumor control in 5 eyes of 5 different patients, these 5 eyes did not need subsequent IA chemotherapy. However, no patient was cured by intravenous carboplatin and local treatments only, and 14 eyes in the 11 patients needed additional IA chemotherapy. All 7 Reese-Ellsworth Group V eye required IA chemotherapy as none was cured with IV chemotherapy alone. Of the 12 Reese-Ellsworth Group I-IV eyes, 5 were cured with IV chemotherapy while 7 required additional IA chemotherapy. One eye was enucleated (patient #1) due to progression of disease after catheterization failure. The other 18 eyes are in place with regressed tumors and regressed seeds. In one eye (patient #3), there was a tumor recurrence 36 months after initial treatment, which was retreated with one additional IA chemotherapy procedure and laser. Figure 2 is an example of bilateral treatment. No radiation therapy has been given. For the 19 eyes, average ocular survival from start of treatment was 24 months (range 8–46 months), and the Kaplan Meier estimate of ocular radiation-free survival was 94.7% at one year (95% confidence interval 68.1–99.2%).

ERGs were measured during the first EUA before any treatment, during and after treatment. ERG results are detailed in Table 1. The final ERG was “good”, “very good” or “excellent” in 14 eyes and “poor” or “fair” in 5. No eye with an initial ERG characterized as “good or better”, deteriorated to “poor or fair”; all 5 eyes with final ERG “poor or fair” were “poor or fair” at baseline.

## Discussion

We found that bridge IV-IA chemotherapy was feasible and safe, and is a promising strategy to treat retinoblastoma in neonates and young infants. Although most (18/19) eyes were preserved, longer follow-up is necessary because recurrences and appearance of new tumors are common in young children. Furthermore, patients 10 and 11 have short follow-up after last treatment. So far, no patient has received external beam radiation therapy, and at least one eye was preserved in all children with bilateral retinoblastoma. Using ERG as a surrogate for visual potential, 14 of the 19 preserved eyes have good vision potential (75.1 µV or greater). In the first patient, the fourth catheterization through the ophthalmic artery failed, resulting in tumor growth and enucleation. This was early in our experience, and if this eye were treated now, we would have used the alternate technique with transient balloon occlusion of the internal carotid artery to perform more IA chemotherapy, and maybe we would have been able to save this eye also.

Our small patient group is representative of early-diagnosed retinoblastoma: at presentation, 10/20 eyes were classified as Group I–III in the Reese-Ellsworth classification, while the other 10 eyes had advanced tumors classified as Group IV–V, confirming that “detection at early age does not guarantee early stage” [1]. Ten of the 11 children had the genetic form of the disease (9 bilateral retinoblastoma and one unilateral patient with positive family history); thereby confirming that children with early-diagnosed retinoblastoma most often have the genetic form of the disease [2].

Preserving vision is especially challenging in early-diagnosed retinoblastoma: the tumors are often in a posterior location -close to the optic disc and the macula- where extensive local treatments such as cryotherapy, laser or radioactive plaque would permanently compromise vision [19]. Intravenous chemotherapy is very effective in shrinking the tumors when performed in conjunction with local treatments, but may be less effective in children younger than 2 months of age [20]. Finally, external beam radiation is no longer given in children younger than one year of age due to the risks of secondary cancers, as well as orbital and midfacial dysplasia [4]. Careful follow-up is especially important owing to risk of developing new tumors in early diagnosis and hereditary cases: 58% risk if age of diagnosis is 0–3 months and 39% if 3–6 months [2]. However, these new tumors appear in a location more anterior to the original tumors, and with monthly follow-up, they can be diagnosed early when their size is still limited, permitting local treatment.

We think that IA chemotherapy greatly improves the treatment of retinoblastoma, but we have hesitated to treat the youngest children, as IA chemotherapy is an invasive technique that requires repeated placements of an arterial sheath in the femoral artery and catheterizations of the ophthalmic artery. Our direct microcatheterization technique, which consists of advancing the microcatheter from the femoral sheath to the ophthalmic artery without the help of a larger intermediary catheter, allows for reduction in the size of the femoral access sheath to 1.3 mm diameter instead of the usual 2.5 mm. Still, in neonates and young infants, where these arteries are just slightly larger than the catheters, catheterization might not be successful or may result in complications, such as arterial thrombosis or dissection. Vasospasm might also be an issue, although it is usually enough to wait for its resolution before pursuing catheterization; on the contrary, atherosclerosis, the largest cause of neurological complication in adults due to emboli from atherosclerotic plaques, is not an issue in young children. Finally, the current drug of choice for IA chemotherapy (melphalan) may induce significant neutropenia when given at a dose higher than 0.4 mg/kg [11]. It is feared that the smallest eye dose of melphalan (2.5 mg) may be toxic systemically when given to children weighing less than 6 Kg. While somewhat arbitrary, our threshold of 3 months and 6 Kg for switching from intravenous to IA chemotherapy represents a compromise between potential risks and benefits, and we will not hesitate to advance or postpone the start of IA chemotherapy according to the tumor staging and the efficacy and tolerance of intravenous chemotherapy.

For the intravenous component of the bridge therapy, we have used single agent carboplatin rather than multi-agent chemotherapy (as other centers might have used) because it was intended to act as a bridge to IA chemotherapy and was not aimed to be curative. Our previous experience with single-agent carboplatin had shown that, although it appeared to be associated with inferior ocular event-free survival than tri-therapy together with vincristine and etoposide, the response rate was comparable and it was generally well tolerated [21]. The potential benefits of using just a few cycles of single agent intravenous carboplatin followed by IA chemotherapy versus six cycles of triple chemotherapy with vincristine, carboplatin, and etoposide are multiple: less risk of ototoxicity [22], etoposide induced leukemia [23], failure to thrive, admission for antibiotics for an infected port or systemic infections, and transfusion of blood products [24]–[26]. The disadvantage of this approach is that some tumors might have been cured with multiagent intravenous chemotherapy without having to use IA chemotherapy. We felt that the reduced toxicity and lack of need for central venous catheter placement outweighed the possible increased efficacy of multi-agent chemotherapy, since we hypothesized that the subsequent IA chemotherapy would make our combination highly effective.

For the intra-arterial component of the bridge therapy, we used the drugs and dosage previously described by our group [8], [9], [11]. Dosage was adjusted at each treatment according to the result of the ophthalmic examination and the retinal tolerance suggested by the electroretinogram. Melphalan 2.5–3 mg was the first choice, and was supplemented with topotecan 0.3–0.5 mg in cases of insufficient tumor regression, or straightaway if severe vitreous seeding. Carboplatin 25–30 mg was used as an alternative to melphalan when performing bilateral simultaneous infusions where the contralateral eye received melphalan, due to concerns of systemic toxicity from a double dose of melphalan; in that case, carboplatin was used alone or supplemented with topotecan 0.3–0.5 mg in cases of insufficient tumor regression, or straightaway if severe vitreous seeding. Finally, tritherapy associating melphalan carboplatin and topotecan was used in the most difficult cases [8], [11].

In conclusion, although these results are preliminary and more follow-up is needed, bridge IV-IA chemotherapy seems to potentiate the advantages and decrease the disadvantages of both intravenous and IA chemotherapy in the youngest children with retinoblastoma: initial single agent intravenous chemotherapy acts to bridge the time from diagnosis to when threshold criteria are met for IA chemotherapy. It allows for the initiation of treatment while invasive IA chemotherapy is postponed, and avoids the potential toxicity of multi-agent chemotherapy. Then, and if necessary, the more effective IA chemotherapy can be performed more easily and safely when the child is older and bigger.
